# The Impact of Preoperative Nicotine Use in the Development of Opioid Use Disorder Following Lumbar Disc Discectomy Procedures: A National Database Study

**DOI:** 10.7759/cureus.91511

**Published:** 2025-09-02

**Authors:** Jinpyo Hong, Andrew Kim, Zachary Freedman, Shoshanna Jadoonanan, David R Hallan, Elias Rizk, John P Kelleher

**Affiliations:** 1 Neurological Surgery, Penn State University College of Medicine, Milton S. Hershey Medical Center, Hershey, USA; 2 Anesthesiology, Beth Israel Deaconess Medical Center, Harvard Medical School, Boston, USA; 3 Neurological Surgery, Dell Medical School, University of Texas at Austin, Austin, USA

**Keywords:** lumbar disc discectomy, nicotine, opioid, opioid use disorder, postoperative outcomes

## Abstract

Background: Opioid prescriptions are highly prevalent for patients undergoing lumbar discectomy procedures for pain control. Management is further complicated in patients with preoperative substance abuse, a known risk factor for addiction. The purpose of this study is to determine whether preoperative nicotine use influences the risk of developing opioid use disorder (OUD) following lumbar discectomy procedures.

Methods: The TriNetX Research database was retrospectively analyzed to identify subjects in the U.S. who underwent lumbar discectomy and received postoperative opioid treatment within two weeks of the procedure. Individuals were then divided into two cohorts based on the presence of nicotine dependence prior to surgery (nicotine dependent vs. non-nicotine dependent) and propensity score matched on known risk factors of addiction, with a matched cohort sample size of 18,305. Development of OUD was then assessed at one, three, six, 12, and 36 months after surgery. The secondary surgical outcomes, including hematoma and surgical site infection (SSI), were also examined. Additional outcomes included myocardial infarction (MI), pulmonary embolism (PE)/venous thromboembolism (VTE), and emergency department (ED) visits one year after surgery.

Results: Nicotine dependence was associated with an increased diagnosis of OUD at all time points (36 months: OR 2.533; CI 2.194-2.923). Preoperative nicotine use was also associated with increased risk of SSI (OR 1.218; 1.033-1.437). Myocardial infarction (12 months: OR 1.698; 1.371-2.102) and ED visits (12 months: OR 1.506; 1.433-1.583) were significantly increased at all time points. There was no significant difference between cohorts in developing hematoma or PE/deep vein thrombosis (DVT).

Conclusion: Preoperative nicotine dependence increases the risk of OUD, SSI, MI, and ED visits following lumbar discectomy procedures. Future prospective studies examining the impact of patient education and preoperative smoking cessation programs are warranted to evaluate their impact on postoperative outcomes.

## Introduction

In the U.S., smoking remains the leading cause of preventable disease, disability, and death [1]. Cigarette smoking is associated with various health problems, including poor bone health. Specifically, smoking can lead to an increased risk of osteoporotic fractures and delayed bone healing [2]. Chronic cigarette smoking can result in mineral-deficient vertebrae that are weak, have impaired blood supply, and contain a reduced number of osteoblasts [3]. Smoking is also associated with adverse postoperative surgical outcomes following lumbar discectomy procedures, including increased risks of postoperative infection, septicemia, pneumonia, recurrence of lumbar disc herniation, and revision discectomy [4-6]. Several mechanisms contribute to the recurrence of lumbar disc herniation in smokers who have undergone discectomy. Smoking inhibits nutritional diffusion into intervertebral discs by contracting capillary vessels that are detached from the bone-disc junction [7]. Additionally, nicotine may be directly responsible for intervertebral disc degeneration through damage to the nucleus pulposus and annulus fibrosus, leading to inhibition of cell proliferation [8]. 

Opioids are a group of analgesic agents that act on opioid receptors [9]. They are commonly used in clinical practice for pain relief following lumbar discectomy procedures; however, they have a high potential for abuse. Among individuals who develop persistent prescription use of opioids, spine-related disorders are among the most common causes for the initial prescription [10]. Gerbershagen et al. reportedly found that in a study of 179 surgical procedures, spinal surgery was among those that caused the highest postoperative pain levels in patients [11].

Opioid abuse has been deemed a public health crisis in the U.S. Opioid abuse may result in opioid use disorder (OUD), which is defined as chronic opioid use resulting in clinically significant distress or impairment. This disorder is associated with repeated craving or desire for opioids, increased opioid tolerance, and withdrawal syndrome when discontinued [12]. As surgeons are among the most frequent prescribers of opioids, there is a need to identify risk factors for sustained postoperative opioid use [13]. Among the most common risk factors for sustained postoperative opioid use is preoperative opioid use [14-17]. Additional risk factors include a history of smoking, advanced age, anxiety, depression, drug abuse, and 30-day readmission [15,16].

The mechanisms by which nicotine use influences sustained postoperative opioid use are not fully understood. This retrospective study aimed to evaluate whether preoperative nicotine dependence increases the risk of developing OUD after lumbar discectomy by employing a population-based relative risk analysis through a national database. This study also aims to understand the differences in risks of developing nine additional secondary postoperative outcomes between the two cohorts.

## Materials and methods

Data source

A retrospective cohort study utilizing the TriNetX Network research database was performed. TriNetX is a multi-institutional, deidentified research cohort derived from electronic medical records from 84 healthcare systems across the U.S. that allows for query-based data extraction based on International Classification of Diseases (ICD-10) and Current Procedural Terminology (CPT) codes. Studies utilizing this database are exempt from Institutional Review Board approval. Penn State University has an agreement in place to access the TriNetX research network.

Study design

The CPT codes were used to identify patients who underwent lumbar discectomy procedures and received postoperative opioid treatment within two weeks of the operation. Subjects were then divided into two cohorts based on whether they had a preoperative diagnosis of nicotine dependence (nicotine dependent and non-nicotine dependent). The primary outcome was new-onset OUD. Secondary outcomes included myocardial infarction (MI), pulmonary embolism (PE)/venous thromboembolism (VTE), upper respiratory tract infection (URI), development of hematoma, surgical site infection, intraoperative complication, postprocedural complication, and emergency department (ED) visit less than one month postoperatively. The ICD-10 and CPT codes utilized are listed in Tables 1-2.

**Table 1:** CPT terminology of lumbar discectomy procedure CPT: Current procedural terminology

CPT terminology	Code
Excision of thoracolumbar vertebral disc, open approach	0RBB0ZZ
Excision of thoracolumbar vertebral disc, percutaneous approach	0RBB3ZZ
Excision of thoracolumbar vertebral disc, percutaneous endoscopic approach	0RBB4ZZ
Resection of thoracolumbar vertebral disc, open approach	0RTB0ZZ
Excision of lumbar vertebral disc, open approach	0SB20ZZ
Excision of lumbar vertebral disc, percutaneous approach	0SB23ZZ
Excision of lumbar vertebral disc, percutaneous endoscopic approach	0SB24ZZ
Excision of lumbosacral disc, open approach	0SB40ZZ
Excision of lumbosacral disc, percutaneous approach	0SB43ZZ
Excision of lumbosacral disc, percutaneous endoscopic approach	0SB44ZZ
Resection of lumbar vertebral disc, open approach	0ST20ZZ
Resection of lumbosacral disc, open approach	0ST40ZZ

**Table 2:** ICD-10 codes of matched diagnoses ICD: International classification of diseases

ICD-10 diagnosis	Code
Alcohol disorder	F10
Benzodiazepine disorder	F13
Depressive disorder	F32
Anxiety disorder	F41
Heart failure	I50
Chronic obstructive pulmonary disease (COPD)	J44

Cohort comparison

The study participants were reviewed for preoperative comorbidities and matched based on the following factors: age, sex, race, ethnicity, depression, anxiety, alcohol use disorder, and benzodiazepine use disorder. Information on race was collected from electronic health record (EHR) reports, and we included this variable to examine for potential health inequities and/or disparities among patients undergoing lumbar discectomy procedures and their risks of developing OUD. Factors used for matching were selected from a prior study [18]. Matching methodology was performed using nearest neighbor algorithms with a caliper width of 0.1 pooled standard deviation. Data was analyzed using the TriNetX software (TriNetX LLC, Cambridge, MA, USA), which utilizes the JAVA (Oracle Corp., Santa Clara, CA, USA), R (R Foundation for Statistical Computing, Vienna, AUT), and Python (Python Software Foundation, Wilmington, DE, USA) programming languages. The p-values for discrete or categorical variables were calculated using a Z-test, while p-values for continuous variables were calculated using a T-test. Statistical significance was set at a p-value of <0.05 for all analyses.

## Results

Our initial search identified 19,627 and 76,202 patients for the nicotine-dependent and non-nicotine-dependent cohorts, respectively. After propensity score matching, both cohorts had 18,305 patients. The average ages for the nicotine-dependent and non-nicotine-dependent cohorts were 50.42 and 49.89 years, respectively. Table 3 summarizes patient characteristics and demographics pre-propensity score matching. Table 4 summarizes patient characteristics and demographics post-propensity score matching.

**Table 3:** Pre-propensity score-matching baseline characteristics and demographics of nicotine-dependent and non-nicotine-dependent patient cohorts An alpha level of 0.05 was selected for statistical significance. *The p-values for discrete or categorical variables were calculated using a Z-test, while p-values for continuous variables were calculated using a T-test. SD: Standard deviation; COPD: Chronic obstructive pulmonary disease

Baseline characteristic	Nicotine use	Percentage of cohort	No nicotine use	Percentage of Cohort	p-value*
Patients (n)	19,627	100%	76,202	100%	-
Age at index (mean ± SD)	50.42 ± 12.87	100%	56.33 ± 15.23	100%	<0.0001
Male (n)	10,855	55.31%	37,998	49.87%	<0.0001
Female (n)	8,771	44.69%	38,124	50.03%	<0.0001
White (n)	13,962	71.14%	52,960	69.50%	<0.0001
Black or African American (n)	2,138	10.89%	6178	8.11%	<0.0001
Asian (n)	73	0.37%	952	1.25%	<0.0001
American Indian or Alaska Native (n)	56	0.29%	337	0.44%	0.002
Native Hawaiian or Other Pacific Islander (n)	18	0.09%	41	0.05%	0.056
Unknown race (n)	3,380	17.22%	15734	20.65%	<0.0001
Not Hispanic or Latino (n)	15,732	80.16%	55569	72.92%	<0.0001
Hispanic or Latino (n)	673	3.43%	3161	4.15%	<0.0001
Unknown ethnicity (n)	3,222	16.42%	17472	22.93%	<0.0001
Depression (n)	5,128	26.13%	9400	12.34%	<0.0001
Anxiety disorder (n)	4,705	23.97%	8528	11.19%	<0.0001
COPD (n)	2,602	13.26%	2208	2.90%	<0.0001
Heart failure (n)	828	4.22%	1931	2.53%	<0.0001
Sedative disorders (n)	202	1.03%	112	0.15%	<0.0001
Alcohol disorders (n)	1,790	9.12%	1043	1.37%	<0.0001

**Table 4:** Post-propensity score-matching baseline characteristics and demographics of nicotine-dependent and non-nicotine-dependent patient cohorts An alpha level of 0.05 was selected for statistical significance. *The p-values for discrete or categorical variables were calculated using a Z-test, while p-values for continuous variables were calculated using a T-test. SD: Standard deviation; COPD: Chronic obstructive pulmonary disease

Baseline characteristic	Nicotine use	Percentage of Cohort	No nicotine use	Percentage of Cohort	p-Value*
Patients (n)	18,305	100%	18,305	100%	-
Age at index (mean ± SD)	50.42 ± 12.93	100%	49.89 ± 14.03	100%	<0.0001
Male (n)	10,074	55.03%	10,175	55.59%	0.288
Female (n)	8,230	44.96%	8,128	44.40%	0.284
White (n)	13,011	71.08%	12,988	70.95%	0.791
Black or African American (n)	1,968	10.75%	1,983	10.83%	0.801
Asian (n)	71	0.39%	79	0.43%	0.513
American Indian or Alaska Native (n)	53	0.29%	34	0.19%	0.041
Native Hawaiian or Other Pacific Islander (n)	14	0.08%	10	0.06%	0.414
Unknown race (n)	3,188	17.42%	3,213	17.55%	0.731
Not Hispanic or Latino (n)	14,549	79.48%	14,629	79.92%	0.299
Hispanic or Latino (n)	650	3.55%	639	3.49%	0.755
Unknown ethnicity (n)	3,106	16.97%	3,037	16.59%	0.335
Depression (n)	4,194	22.91%	4,287	23.42%	0.249
Anxiety disorder (n)	3,853	21.05%	3,937	21.51%	0.283
COPD (n)	1,795	9.81%	1,618	8.84%	0.001
Heart failure (n)	654	3.57%	603	3.29%	0.143
Sedative disorders (n)	86	0.47%	77	0.42%	0.480
Alcohol disorders (n)	998	5.45%	881	4.81%	0.006

Nicotine dependence was associated with an increased diagnosis of OUD at one, three, six, 12, and 36 months (36 months: OR 2.533; CI 2.194-2.923). Incidence of MI (12 months: OR 1.698; CI 1.371-2.102) and number of ED visits (12 months: OR 1.506; CI 1.433-1.583) were also significantly increased at one, three, six, and 12 months. Preoperative nicotine dependence was also associated with an increased incidence of surgical site infection (SSI) (OR 1.218; CI 1.033-1.437) at one month (Table 5). All other primary and secondary outcomes are summarized in Table 6 and Table 7, respectively.

**Table 5:** Statistically significantly different postoperative event for the nicotine-dependent and non-nicotine-dependent patient cohorts at the one-month follow-up An alpha level of 0.05 was selected for statistical significance. *The p-values for discrete or categorical variables were calculated using a Z-test, while p-values for continuous variables were calculated using a T-test. CI: Confidence interval; SSI: Surgical site infection

Postoperative event	Time	Odds ratio	95% Cl	p-value*
SSI	1 month	1.218	1.033, 1.437	0.019

**Table 6:** Statistically significantly different primary outcome for the nicotine-dependent and non-nicotine-dependent patient cohorts at one, three, six, 12, and 36 months An alpha level of 0.05 was selected for statistical significance. *The p-values for discrete or categorical variables were calculated using a Z-test, while p-values for continuous variables were calculated using a T-test. CI: Confidence interval; OUD: Opioid use disorder

Primary outcome	Time	Odds ratio	95% Cl	p-value*
OUD	One month	2.527	1.586, 4.027	<0.0001
	Three months	2.667	1.929, 3.689	<0.0001
	Six months	2.726	2.109, 3.525	<0.0001
	One year	2.461	2.011, 3.014	<0.0001
	Three years	2.533	2.194, 2.923	<0.0001

**Table 7:** Statistically significantly different secondary outcomes for the nicotine-dependent and non-nicotine-dependent patient cohorts at one, three, six, and 12 months An alpha level of 0.05 was selected for statistical significance. *The p-values for discrete or categorical variables were calculated using a Z-test, while p-values for continuous variables were calculated using a T-test. CI: Confidence interval; MI: Myocardial infarction; ED: Emergency department

Secondary outcome	Time	Odds ratio	95% Cl	p-value*
MI	One month	1.927	1.373, 2.704	<0.0001
	Three months	1.887	1.425, 2.499	<0.0001
	Six months	1.659	1.295, 2.126	<0.0001
	One year	1.698	1.371, 2.102	<0.0001
ED visit	One month	1.325	1.231, 1.425	<0.0001
	Three months	1.401	1.318, 1.489	<0.0001
	Six months	1.462	1.383, 1.544	<0.0001
	One year	1.506	1.433, 1.583	<0.0001

There was no significant difference between cohorts in developing hematoma or PE/VTE postoperatively. Non-significant postoperative events and secondary outcomes are summarized in Table 8 and Table 9, respectively.

**Table 8:** Non-statistically significantly different post-operative event for the nicotine-dependent and non-nicotine-dependent patient cohorts at the one-month follow-up An alpha level of 0.05 was selected for statistical significance. *The p-values for discrete or categorical variables were calculated using a Z-test, while p-values for continuous variables were calculated using a T-test. CI: Confidence interval; PE: Pulmonary embolism, VTE: Venous thromboembolism

Postoperative event	Time	Odds ratio	95% Cl	p-value*
Hematoma	One month	0.895	0.563, 1.421	0.637

**Table 9:** Non-statistically significantly different secondary outcome for the nicotine-dependent and non-nicotine-dependent patient cohorts at one, three, six, and 12 months An alpha level of 0.05 was selected for statistical significance. *The p-values for discrete or categorical variables were calculated using a Z-test, while p-values for continuous variables were calculated using a T-test. CI: Confidence interval; PE: Pulmonary embolism; VTE: Venous thromboembolism

Secondary outcome	Time	Odds ratio	95% Cl	p-value*
PE/VTE	One month	0.954	0.820, 1.109	0.537
	Three months	1.025	0.899, 1.169	0.713
	Six months	1.046	0.925, 1.183	0.472
	One year	1.024	0.914, 1.148	0.683

## Discussion

Trends of opioid pain relievers (OPR) have been used to determine the frequency of OUD. The incidence of nonmedical OPR use increased drastically in the late 1990s, accounting for the possible increase in OUD diagnosis among the general population [19]. Along with opioid use, nicotine use was a topic of interest among researchers who studied its effects on surgical outcomes. Limiting preoperative exposure to nicotine for spinal fusion surgery in an animal model study demonstrated an improvement in postoperative results when compared to subjects that had continuous exposure to nicotine [20]. The negative effects of nicotine in the postoperative period seen in animal studies aligned with findings in human studies, as nicotine use was shown to promote vascular calcification and oxidative stress-mediated extracellular vesicle secretion [21].

Our study investigated the effects of nicotine dependence on the development of OUD following lumbar discectomy procedures. The results of our investigation found a statistically significant association between nicotine dependence and increased diagnosis of OUD at all time points. Without considering nicotine dependence as a risk factor, a nationwide retrospective analysis conducted by Idrizi et al. reported that the number of patients with OUD diagnosis who underwent lumbar laminectomy increased over three times from 2005 to 2014 [22]. Furthermore, while nicotine use impacts the diagnosis of OUD postoperatively, Smith et al. also found that the two-year revision rate for smokers was higher when compared to nonsmokers (6.3% vs. 2.5%) [6].

In addition, our study also found an increased risk of SSI with preoperative nicotine use at all time points. This finding contrasts with those found by Blood et al., who found no relationship between SSI and preoperative nicotine use [23]. Their study included cigarette smoking as a risk factor for SSI among orthopedic spinal surgery patients. In their randomized controlled trial, Yang et al. reported results similar to our study, concluding that smoking results in impaired wound healing while increasing risk for wound infections for patients who continued smoking [24].

We also found that preoperative nicotine use was significantly associated with increased risk of MI at all time points. Lau et al. similarly found that among surgical subspecialties, smokers have an increased risk for MI and other cardiovascular complications [25]. Additionally, our study also found that nicotine users were significantly more likely to have more postoperative ED visits compared to non-nicotine users. This finding contrasts with those found by Lee et al., who in their systematic review and meta-analysis stated that active smoking status was not a risk factor significantly associated with unplanned readmission following anterior cervical discectomy and fusion [26]. Our study also found a significant increase in OUD diagnosis following lumbar discectomy, which could be expanded upon by investigating post-diagnosis events, such as length of in-hospital stay and readmission rates. Kim et al. and Gupta et al. found that OUD patients have longer in-hospital stays and 30-day readmission rates in the postoperative period [27,28]. Propensity-score matching between cohorts eliminated a considerable number of confounding variables in our study, given the complexity of OUD diagnoses in patients.

The results of our study should be interpreted in the context of its limitations. The design of this study is retrospective. The research question was formed, and the study was carried out after the index events had already taken place. This points to the nature of retrospective studies, which is embedded with inherent bias. Moreover, the ICD-10 codes that we included in our study could include any inaccurate billing entries that could have led to the inclusion of patients who needed to be excluded and vice versa. Moreover, our study is limited in elucidating clinical relevance from the statistical significance we identified, as these discrete findings do not always extrapolate to clinical settings. Therefore, it has limited generalizability.

Furthermore, our analysis did not incorporate relevant pharmacologic variables such as concurrent sedative use or specific opioid dosing and duration, which further limited the analysis regarding the risks of developing OUD after lumbar discectomy following preoperative use of nicotine. Other pharmacologic confounders, such as gabapentinoids and polypharmacy, were not factored into our study, which are known to influence opioid dependence risk. Omission of social determinants, including but not limited to socioeconomic status and pain scores, also limited our analysis in identifying potential confounders of OUD diagnosis. Lastly, additional confounding variables, such as mental health disorders, were not fully addressed when designing this study.

Clinical-level data were not accessible due to the nature of the database, where the information available within was deidentified and only indicative of the outcome of interest without any additional context for specific patients in different cohorts. Future studies examining the effects of an OUD diagnosis with a follow-up period greater than 36 months through a longitudinal, prospective study are warranted to assess the long-term effects of preoperative nicotine use among patients undergoing lumbar discectomy procedures. Additionally, such studies should stratify postoperative clinical outcomes by organ system. 

## Conclusions

Our study found that there is an increased associated risk of developing OUD, SSI, MI, and ED visits for patients with a preoperative diagnosis of nicotine dependence following lumbar discectomy procedures. Although our main outcome of interest was OUD for this specific study, findings regarding additional adverse postoperative and secondary outcomes warrant individualized patient assessment of overall medical and social history when pursuing lumbar discectomy procedures. Future prospective studies examining the impact that patient education and preoperative smoking cessation programs have on the outcomes of lumbar discectomy procedures will further help us make informed decisions when patient-surgeon discussions take place in the attempt to achieve optimal patient care. In addition, study designs such as a prospective longitudinal cohort study that assess factors including opioid tapering success, readmission rates, and quality of life (QOL) scores will further help characterize the development of OUD in lumbar discectomy patients.
